# Bone turnover biomarkers in COPD patients randomized to either a regular or shortened course of corticosteroids: a substudy of the randomized controlled CORTICO-COP trial

**DOI:** 10.1186/s12931-020-01531-9

**Published:** 2020-10-12

**Authors:** Pradeesh Sivapalan, Niklas R. Jørgensen, Alexander G. Mathioudakis, Josefin Eklöf, Therese Lapperre, Charlotte Suppli Ulrik, Helle F. Andreassen, Karin Armbruster, Praleene Sivapalan, Julie Janner, Nina Godtfredsen, Ulla M. Weinreich, Thyge L. Nielsen, Niels Seersholm, Torgny Wilcke, Philipp Schuetz, Tobias W. Klausen, Kristoffer Marså, Jørgen Vestbo, Jens-Ulrik Jensen

**Affiliations:** 1 Section of Respiratory Medicine, Department of Medicine, Herlev and Gentofte Hospital, University of Copenhagen, Gentofte Hospitalsvej 7, Ground Floor, DK-2900 Hellerup, Denmark; 2 Department of Internal Medicine, Zealand Hospital, University of Copenhagen, Roskilde, Denmark; 3 Department of Clinical Biochemistry, Copenhagen University Hospital Rigshospitalet, Copenhagen, Denmark; 4 Department of Clinical Medicine, Faculty of Health and Medical Sciences, University of Copenhagen, Copenhagen, Denmark; 5 The North West Lung Centre, Wythenshawe Hospital, Manchester University NHS Foundation Trust, Manchester, UK; 6 Division of Infection, Immunity and Respiratory Medicine, School of Biological Sciences, The University of Manchester, Manchester Academic Health Science Centre, Manchester, UK; 7 Department of Respiratory Medicine, Bispebjerg University Hospital, Copenhagen, Denmark; 8 Department of Respiratory Medicine, Amager and Hvidovre University Hospital, Copenhagen, Denmark; 9 Department of Respiratory Diseases, Aalborg University Hospital, Aalborg, Denmark; The Clinical Institute, Aalborg University, Aalborg, Denmark; 10 Department of Respiratory and Infectious Diseases, Frederiksund and Hillerød Hospital, University of Copenhagen, Copenhagen, Denmark; 11 Medical University Department, Kantonsspital Aarau, 5001 Aarau, Switzerland; 12 Faculty of Medicine, University of Basel, 4001 Basel, Switzerland; 13 Clinical Research Unit, Department of Hematology, Herlev Hospital, Herlev, Denmark; 14 Palliative Medicine Section Unit, Herlev and Gentofte Hospital, Herlev, Denmark

**Keywords:** Adverse effects, Bone remodelling, Bone turnover markers, Chronic obstructive pulmonary disease, Corticosteroids

## Abstract

**Background:**

Long-term treatment with corticosteroids causes loss of bone density, but the effects of using short-term high-dose systemic-corticosteroid therapy to treat acute exacerbations of chronic obstructive pulmonary disease (AECOPD) are unclear. Our aim was to determine whether high-dose corticosteroid therapy affected bone turnover markers (BTMs) to a greater extent compared to low-dose corticosteroid therapy.

**Methods:**

The CORTICO-COP trial (NCT02857842) showed that an eosinophil-guided corticosteroid intervention led to approximately 60% lower accumulated corticosteroid dose for hospitalized patients with AECOPD (low-dose group) compared with 5-day standard corticosteroid treatment (high-dose group). We compared the levels of BTMs C-terminal telopeptide of type 1 collagen (CTX) and procollagen type 1 N-terminal propeptide (P1NP) in 318 participants during AECOPD and at 1- and 3-month follow-up visits.

**Results:**

CTX decreased and P1NP increased significantly over time in both treatment groups. There were no significant differences between the groups at 1- or 3-months follow-up for P1NP. A significant drop in CTX was seen at 3 months (down Δ24% from the baseline, *p* = 0.017) for the high dose group.

**Conclusion:**

Short-term, high-dose systemic corticosteroid treatment caused a rapid suppression of biomarkers of bone resorption. Corticosteroids did not suppress biomarkers of bone formation, regardless of patients receiving low or high doses of corticosteroids. This therapy was, therefore, harmless in terms of bone safety, in our prospective series of COPD patients.

**Trial registration:**

ClinicalTrials.gov Identifier: NCT02857842. Submitted August 2nd, 2016.

## Background

Acute exacerbation of chronic obstructive pulmonary disease (COPD) can lead to hospitalization and is then associated with an increased risk of mortality [1]. Rescue courses of systemic corticosteroids are the standard of care for more severe acute exacerbations of COPD (AECOPD). Certain patients with COPD experience frequent exacerbations and are, therefore, exposed to repeated courses of corticosteroids for 5–7 days [2]. Patients with COPD are at increased risk of osteoporosis and fragility fractures as compared to healthy individuals [3]. Corticosteroids have both anabolic and catabolic effects on bone. However, overall, corticosteroid therapy results in decreases in bone formation and bone mineral density and an increase in bone resorption, as well as higher risks of fractures and avascular necrosis [4–6]. Corticosteroid-induced osteoporosis is the most common form of secondary osteoporosis, and the incidence of fractures in patients receiving long-term corticosteroid therapy is as high as 30–50% [7]. For patients undergoing chronic treatment with corticosteroids, the major mechanism of bone loss is decreased bone formation due to reductions in the osteoblastic pool and osteoblast differentiation, especially during the early phase [8]. Corticosteroid administration may prevent osteoclast development from macrophages and limit cytoskeletal function in a dose-dependent manner [9].

Bone remodelling may be assessed using biochemical markers that serve as indicators of overall skeletal bone formation by osteoblasts and bone resorption by osteoclasts [10]. Decreased bone formation can be clinically evaluated by monitoring decreases in the circulating concentration of the bone formation marker procollagen type 1 N-terminal propeptide (P1NP). Similarly, bone degradation can be assessed by monitoring increases in the bone resorption marker C-terminal telopeptide of type 1 collagen (CTX) [11].

Many studies have investigated loss of bone density in patients undergoing chronic treatment with systemic corticosteroids, but most of these studies involved patients who had long-term treatment with various doses of corticosteroids [12, 13]. In contrast, little is known about the effects on bone metabolism of short-term therapy with high doses of corticosteroids, such as in the treatment of AECOPD [14]. Overall, the effects of corticosteroids on bone metabolism are not well characterised [15]. Our primary aim of this study was to explore the adverse effects of corticosteroids on bone by evaluating the change in bone turnover markers (BTMs) over time after AECOPD [16].

This randomized controlled trial (RCT) substudy evaluated the effects of two different corticosteroid dosing regimens (“low dose” vs. “high dose”) on BTMs in patients with AECOPD over a 3-month follow-up period.

## Methods

### Study design and patients

CORTICOsteroid reduction in COPD (CORTICO-COP) was a 3-month, investigator-initiated, open-label, randomized, non-inferiority trial (clinical trials.gov NCT02857842) of eosinophil-guided corticosteroid treatment compared with 5-day standard corticosteroid treatment for patients with AECOPD [17]. The eosinophil-guided corticosteroid treatment led to patients receiving an approximately 60% lower accumulated dose of systemic corticosteroids; thus, this arm of the trial was named the “low-dose group” in the current study, whereas the control arm was named the “high-dose group” (Table 1). We investigated changes in the levels of BTMs from baseline to 3 months follow-up in these two treatment groups. CTX and P1NP measurements were recorded from 318 patients during AECOPD and at 1- and 3-month visits. This study was approved by the Ethics Committees of all participating sites (H-15012207) and the Danish Data Protection Agency (HGH-2015-038 and I-Suite number 04014).

**Table 1:** Baseline characteristics

	Low-dose group ***N*** = 159	High-dose group ***N*** = 159
**Age [years]**	75 (69–81)	75 (68–82)
**Females,** ***n*** **(%)**	86 (54%)	89 (56%)
**BMI [kg/m**^**2**^**]**	24.2 (20.8–26.6)	23.6 (20.3–27.9)
**FEV**_**1**_**% predicted**	32 (23.0–39)	30 (23.0–40.5)
**Severe exacerbation rate in the previous 12 months, mean (95% confidence interval)**	0.64 (0.45–0.83)	0.69 (0.44–0.94)
**Smokers,** ***n*** **(%)**	54 (34%)	50 (31%)
**Pack years**	45 (30–57)	45 (30–57)
**Ca**^**2+**^ **[mmol/L]**	1.18 (1.15–1.22)	1.19 (1.15–1.22)
**25OHD3 [nmol/L]**	76 (48–103)	87 (58–111)
**PTH [ρmol/L]**	5.4 (4.1–7.6)	5.4 (4.8–7.4)
**Fasting blood glucose [mmol/L]**	8.1 (7.6–8.6)	8.0 (7.6–8.4)
**Leucocytes [× 10**^**9**^**/L]**	9.8 (7.5–13.5)	9.9 (7.9–13.1)
**CRP [mg/L]**	22 (8–70)	33 (11–104)
**Corticosteroids during hospitalisation [days]**	2 (1–3)	5 (5–5)
**Cumulative corticosteroid dose during hospitalisation (mg)**	121 (113–130)	225 (222–228)
**Cumulative corticosteroid dose during 3-month follow-up (mg)**	261 (216–301)	421 (353–488)
**Inhaled corticosteroid therapy before recruitment**	80 (50%)	96 (60%)
**Prednisolone prescription 2 weeks before recruitment,** ***n*** **(%)**	8 (5%)	12 (8%)
**Maintenance of prednisolone treatment (≤ 10 mg) daily,** ***n*** **(%)**	10 (6%)	7 (4%)
**Osteoporosis,** ***n*** **(%)**	33 (21%)	26 (16%)
**Bisphosphonates within the 12 months before inclusion,** ***n*** **(%)**	18 (11.3%)	15 (9.4%)
**Denosumab within the 12 months before inclusion,** ***n*** **(%)**	8 (5.0%)	2 (1.3%)
**Synthetic human parathyroid hormone within the 12 months before inclusion,** ***n*** **(%)**	1 (0.6%)	0

Data are expressed as medians (interquartile ranges) unless otherwise stated

Abbreviations: *BMI* body mass index, *FEV*_*1*_ forced expiratory volume in 1 s, *PTH* parathyroid hormone, *Ca*^*2+*^ calcium ion concentration, *25OHD3* 25-hydroxyvitamin D3, *CRP* C-reactive protein

### Biochemical measurements

Serum calcium metabolic parameters, including 25-hydroxyvitamin D3 (25OHD3), parathyroid hormone (PTH) and calcium concentrations, were measured before commencing corticosteroid therapy. Vitamin D supplements were given to patients with low serum levels (< 50 nmol/L) of 25OHD3, and calcium supplements were given to patients who had a high risk of osteoporosis or frequent corticosteroid courses in the past. CTX and P1NP samples were centrifuged immediately after collection and stored at − 80 °C until they were analysed. Fasting blood samples were collected at baseline between 7 am and 9 am, after fasting for at least 12 h. It was not always possible to collect 1- and 3-month fasting blood samples because patients had difficulty attending study visits in the morning. Plasma CTX levels were measured using the IDS-iSYS CTX CrossLaps® assay (Immunodiagnostic Systems, Tyne and Wear, UK). Plasma P1NP levels were measured using the IDS-iSYS intact P1NP assay (Immunodiagnostic Systems). Both assays were chemiluminescence immunoassays and were carried out on an iSYS automated analyser (Immunodiagnostic Systems) according to the manufacturer’s instructions. None of the samples had been previously thawed, and all analyses were performed immediately after thawing the samples. All samples analysed were from a single batch in each assay. Assay performance was verified using the manufacturer’s control specimens. The iSYS intermediary precisions expressed as coefficients of variation were 5.3, 3.4 and 3.5% for CTX concentrations of 213 ng/L, 869 ng/L and 2113 ng/L, respectively. The iSYS intermediary precisions were 5.4, 6.5 and 6.1% for P1NP concentrations of 18.96 μg/L, 48.48 μg/L and 122.10 μg/L, respectively. Total 25OHD3 was measured directly using a competitive immunoassay. Calcium and fasting blood glucose concentrations were measured using standard methodologies.

### Statistical analysis

Data are expressed as medians with interquartile ranges unless otherwise stated. A *p*-value < 0.05 was considered statistically significant. Logarithmic transformation was used to normalise non-normal distributions. To investigate the sensibility of the log transformation, the dependent variable was transformed according to the Box-Cox power transformations. The mixed model used for calculations were performed using CTX and P1NP transformed by different lambda values using maximum likelihood estimation and the log-likelihoods were calculated depending on lambda value [18]. Furthermore, the residuals were visually examined using histograms and qq-plots (Supplementary material, Figure 3S). Constrained longitudinal data analysis was used to evaluate the effects of corticosteroids on baseline levels of P1NP and CTX. The advantage of this analysis is that it includes baseline measurements as dependent variables [19]. After randomization, the frequency of active treatment for osteoporosis was apparently skewed. Therefore, we also performed sensitivity analyses, excluding patients who had received appropriate treatments within the 12 months before inclusion. We also performed post hoc analyses looking at the linear association between BTMs and cumulated corticosteroids during the 1- and 3-month follow-up (Supplementary material, Table 1S). Finally, stratified analysis on inhaled corticosteroid (ICS) use vs. no ICS use was performed (Supplementary material, Table 2S) for the BTMs. Statistical analyses were performed using SAS software (ver. 9.4; SAS Institute, Inc., Cary, NC, USA).

## Results

**Fig. 1:**
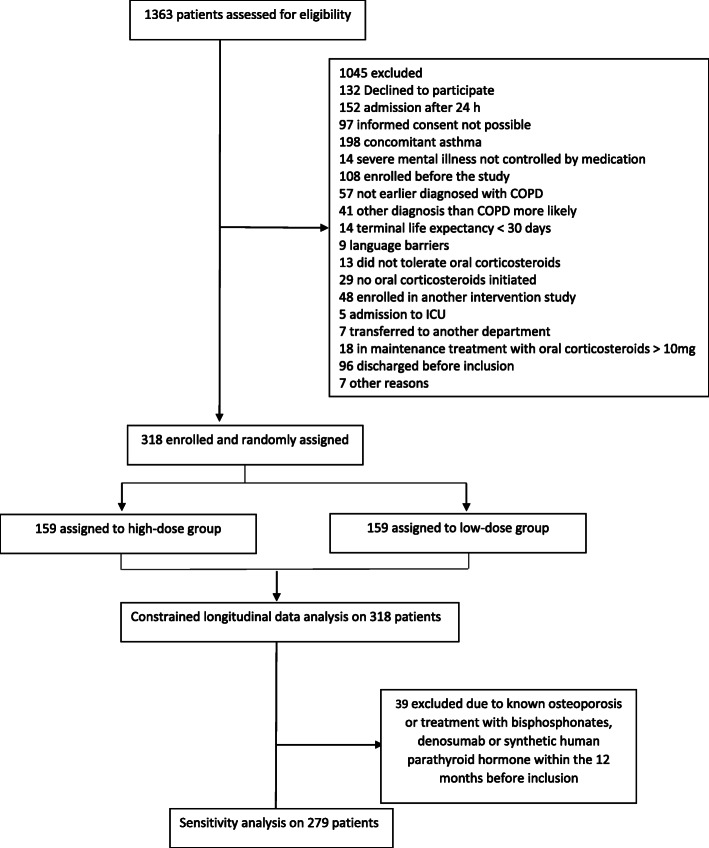
CONSORT flowchart

Between August 2016 and September 2018, 318 patients were enrolled in the CORTICO-COP randomized controlled trial; Fig. 1 [17]. There were no significant differences between the treatment groups in terms of calcium, 25OHD3 or PTH concentrations at the time of recruitment (Table 1). Approximately 50% of the patients in each group were on ICS treatment (Table 1). The median times for blood samplings were 06:52 (06:13 - 07:25), 10:11 (09:00 - 12:03) and 10:30 (9:25 - 12:36) for AECPOD, 1-month follow up and 3 months follow-up, respectively. The percentages of patients who had fasting blood samples were 83.1 % vs. 89.6% (*p* = 0.11) at the time of AECOPD, 3.8% vs. 7.0% on day 30 (*p* = 0.35) and 1.9% vs. 6.3% (*p* = 0.07) at day 90. (Supplemental material Table 3S).

**Fig. 2:**
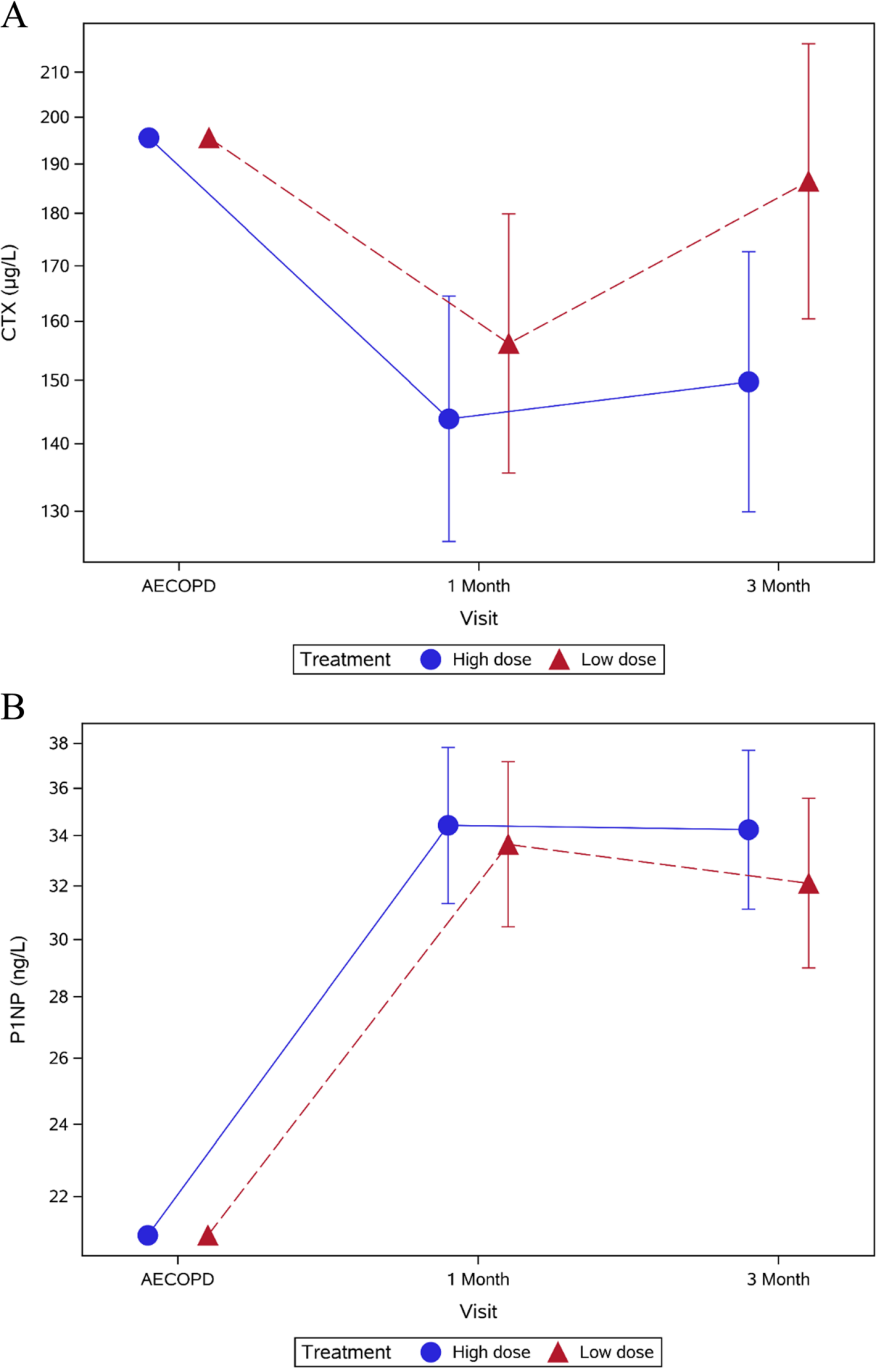
**a** CTX and **b** P1NP measurements for the low- and high-dose groups 3 months after AECOPD. Error bars indicate 95% confidence interval

CTX levels declined significantly from baseline to 3 months in both the low-dose and high-dose groups (Fig. 2). There was no difference in CTX levels between the treatment groups at 1 month: relative difference at 1 month = 9% (− 8, 28%; *p* = 0.33). However, at 3 months a significant drop in CTX was seen for the high dose group: relative difference = 24% (4, 49%); *p* = 0.017; Table 2).

**Table 2:** Percentage change in CTX and P1NP levels from baseline (AECOPD) to 1- and 3-month follow-up

Total *N* = 318	Baseline (μg/L) *N* = 298	1 month (difference from baseline (95% CI)) ***N*** = 213	3 months (difference from baseline (95% CI)) ***N = 193***
**CTX** **Low-dose group**	196	−20% (− 30, − 8%); *p* < 0.0013	− 4% (− 17, 10%); *p* = 0.55
**CTX** **High-dose group**	196	−26% (− 35, − 16%); *p* < 0.0001	−23% (− 32, − 12%); *p* = 0.0001
**Relative difference between treatment groups**^**a**^		9% (−8, 28%); *p* = 0.33	24% (4, 49%); *p* = 0.017
**P1NP** **Low-dose group**	34	60% (47, 75%); *p* < 0.0001	53% (38, 69%); *p* < 0.0001
**P1NP** **High-dose group**	34	64% (51, 78%); *p* < 0.0001	63% (49, 79%); *p* < 0.0001
**Relative difference between treatment groups**^**a**^		−2% (− 14, 10%); *p* = 0.70	−6% (− 18, 7%); *p* = 0.33
P1NP/CTX ratio Low dose group	0.11	2.18 (1.91–2.49) *p* < 0.0001	2.11 (1.82; 2.40) *p* < 0.0001
P1NP/CTX ratio High dose group	0.11	2.08 (1.81;2.39) *p* < 0.0001	1.70 (1.46; 1.97) *p* < 0.0001
**Relative difference between treatment groups**^**a**^		0.95 (0.81; 1.12) *p* = 0.56	0.81 (0.67; 0.98) *p* = 0.029

^a^the relative difference between groups is calculated as the time–group interaction at the specific visit

Abbreviations: *CTX* C-terminal telopeptide of type 1 collagen, *P1NP* procollagen type 1 N-terminal propeptide

Similarly, P1NP increased significantly from baseline to 3 months in both groups. There was no difference in P1NP levels between the treatment groups at either time point: relative difference at 1 month = − 2% (− 14, 10%; *p* = 0.70); relative difference at 3 months = − 6% (− 18, 7%; *p* = 0.33; Table 2, Fig. 2). The P1NP/CTX ratio increased significantly from baseline to 1- and 3-months follow-up (Tables 2 & 3). However, we only found a difference between the treatment groups at 3-months follow-up for the primary analysis (Table 2).

**Table 3:** Percentage change in CTX and P1NP levels from baseline (AECOPD) to 1- and 3-month follow-up excluding patients who had osteoporosis and those who had taken bisphosphonates, denosumab or synthetic human parathyroid within the 12 months before inclusion

Total *N* = 279	Baseline (μg/L) *N* = 264	1 month (difference from baseline (95% CI)) ***N*** = 193	3 months (difference from baseline (95% CI)) ***N = 179***
**CTX** **Low-dose group**	257	− 26% (− 36, − 14%); *p* = 0.0002	−14% (− 27, 1%); *p* = 0.063
**CTX** **High-dose group**	257	−32% (− 40, − 22%); *p* < 0.0001	− 30% (− 40, − 19%); *p* < 0.0001
**Relative difference between treatment groups**^**a**^		8% (−10, 31%); *p* = 0.40	23% (0, 51%); *p* = 0.049
**P1NP** **Low-dose group**	22	65% (49, 83%); *p* < 0.0001	55% (38, 73%); *p* < 0.0001
**P1NP** **High-dose group**	22	62% (48, 78%); *p* < 0.0001	60% (44, 78%); *p* < 0.0001
**Relative difference between treatment groups**^**a**^		2% (−11, 16%); *p* = 0.80	−4% (− 17, 12%); *p* = 0.63
P1NP/CTX ratio Low dose group	0.10	2.29 (1.98–2.65) *p* < 0.0001	2.20 (1.89; 2.56) *p* < 0.0001
P1NP/CTX ratio High dose group	0.10	2.22 (1.89–2.60) *p* < 0.0001	1.80 (1.53; 2.12) *p* < 0.0001
**Relative difference between treatment groups**^**a**^		0.97 (0.81; 1.17) *p* = 0.74	0.82 (0.67; 1.00) *p* = 0.052

^a^the relative difference between groups is calculated as the time–group interaction at the specific visit

Abbreviations: *CTX* C-terminal telopeptide of type 1 collagen, *P1NP* procollagen type 1 N-terminal propeptide

### Sensitivity analyses

Excluding 39 patients with known osteoporosis and those who had taken bisphosphonates, denosumab or synthetic human parathyroid hormone within the 12 months before inclusion did not change our results for the BTM levels. The change in CTX levels at 3 months follow-up was − 14% (− 27, 1%) for the low-dose group and − 30% (− 40, − 19%) for the high-dose group. The relative difference between these treatment groups at 3 months was 23% (0, 51%; *p* = 0.049; Table 3). The increase in P1NP levels at 3 months follow-up was 55% (38, 73%) for the low-dose group and 60% (44, 78%) for the high-dose group. The relative difference between these treatment groups was − 4% (− 17, 12%; *p* = 0.63; Table 3).

We also performed stratified analysis, according to whether patients were on ICS treatment. We observed an increased CTX value at 3 months in the low dose group for patients receiving ICS (37.4% (10.2, 71.4%; *p* = 0.005)) compared to patients not receiving ICS. For P1NP there was no difference in BTMs between the groups when stratified for ICS at any time point (supplementary material, Table 2S).

## Discussion

In hospitalized patients with AECOPD who, by random allocation, received either low- or high accumulated dose of systemic corticosteroid treatment we found that CTX levels declined and P1NP levels increased between baseline and 3 months in both groups. We did find lower CTX levels in the high dose group compared to the low dose groups at 3-month follow up. The increased PINP / CTX ratio suggests that there is a better balance between formation and resorption measured by the respective biomarkers during follow-up.

To date no other publications have been devoted to the assessment of corticosteroid effects on BTMs after AECOPD. The decreases in CTX levels observed in both treatment groups over time could be related to reduced inflammation and recovery after an exacerbation episode. Studies on patients with rheumatoid arthritis has shown increased levels of P1NP and CTX in patients with active disease. Higher concentrations of P1NP and lower concentrations of CTX were observed in short-term corticosteroid treated patients, which is consistent with our results [20]. Furthermore, the CTX levels also decreased with disease progression [21].

Interestingly, we found that CTX levels began to recover after 1 month in both treatment groups, although some patients in both groups received additional corticosteroid courses during the follow-up period. This trend in serum CTX levels was also observed in other studies [15, 22] and may reflect the change in dosage or a skeletal adaptation to corticosteroids.

Our findings are consistent with previous studies, that showed that 1 week of treatment with 60 mg of daily prednisone decreased CTX levels, and even lower doses (10 mg of daily prednisone over 1–2 weeks) decreased CTX levels by 10% [23]. Another study investigating the effects of short-term, high-dose corticosteroid treatment on BTMs reported a significant decrease in CTX levels 1 month after commencing treatment [22].

Other studies have reported suppression of bone formation in response to long-term treatment with low-moderate doses of prednisolone, which was reflected by decreased P1NP levels [15, 22]. We were not able to reproduce these results in our study. However, P1NP has not been tested in patients with AECOPD. Thus, our results could be due to increased remodelling and increased bone formation after AECOPD.

Treatments for osteoporosis interfere with bone remodelling, and this is reflected by a decrease in the levels of bone resorption BTMs and an increase in the levels of bone formation BTMs [24, 25]. After randomisation, the frequency of active treatment for osteoporosis was apparently skewed. Therefore, we also performed sensitivity analyses, excluding patients who had received relevant osteoporosis treatments within the 12 months before inclusion. Overall, these analyses did not change our results (Table 3).

The strengths of this study included the relatively large sample size and multi-centre RCT design, published treatment protocol [16] and the appropriate selection of biomarkers. Consequently, baseline characteristics in the high- and low-dose groups were similar. Furthermore, all the blood measurements were standardised and performed during baseline and follow-up at three central hospitals.

Although the study has strengths, some limitations deserve careful attention. It was nearly impossible to take fasting blood samples in these severely ill COPD patients during follow-up since they were too ill to come into the hospital early in the morning [26, 27], and this variation may be increased further by food intake [28], which could have affected our analyses at both 1 and 3 months. However, any such effects on the high- and low-dose groups are likely to be similar due to randomisation. The persons who ordered the blood samples at 1 and 3 months, and those who drew the samples, were blinded to high/low dose group allocation. We compared both the time at which blood samples were taken, and the number of fasting blood samples between the two treatment groups and found no differences (Supplemental material (Supplemental material Table 3S). The fact that we did not perform fasting samples at the later time points is certainly a limitation; however, since the error caused by this was randomly distributed between the two arms, we judged that comparisons between these two groups are warranted. In addition, although CTX levels may be affected, PINP levels are less sensitive to food intake. The acute exacerbations may cause systemic inflammation, which, in turn, may affect bone metabolism and our analyses. Furthermore, our results only examine the short-term effects of systemic corticosteroid treatment. For patients who have several AECOPD episodes in a year, the results may vary.

Some of our patients received vitamin D supplements, which could also have influenced our results. However, because this was an RCT, vitamin D supplements were given to both treatment groups and would have had little impact on our results.

## Conclusions

Short-term, high-dose systemic corticosteroid treatment caused a rapid suppression of bone resorption biomarkers. Corticosteroids did not suppress bone formation biomarkers, regardless of patients receiving low or high doses of corticosteroids. This therapy was, therefore, harmless in terms of bone safety, in our prospective series of patients. Further studies are needed to confirm our results, owing to the limitation of our study.

## Supplementary information

**Additional file 1.**

## Data Availability

Data collected for the CORTICO-COP trial, including individual participant data and a data dictionary defining each field in the set, will be made available to others in form of deidentified participant data. The study protocol and statistical analysis plan for the original study is available at www.coptrin.dk. Informed consent forms will not be available according to Danish legislation. These data will become available from Jan 1, 2023, upon request from investigators.

## Abbreviations

AECOPD: Acute exacerbation of chronic obstructive pulmonary disease
BTMs: Bone turnover markers
CRP: C-reactive protein
CTX: C-terminal telopeptide of type 1 collagen
ICS: Inhaled corticosteroids
P1NP: Procollagen type 1 N-terminal propeptide

## References

[1] Wang Q, Bourbeau J (2005). Outcomes and health-related quality of life following hospitalization for an acute exacerbation of COPD. Respirology..

[2] Walters JA, Tan DJ, White CJ, Gibson PG, Wood-Baker R, Walters EH (2014). Systemic corticosteroids for acute exacerbations of chronic obstructive pulmonary disease. Cochrane Database Syst Rev.

[3] Chen YW, Ramsook AH, Coxson HO, Bon J, Reid WD (2019). Prevalence and risk factors for osteoporosis in individuals with COPD: a systematic review and meta-analysis. Chest..

[4] Manolagas SC, Weinstein RS (1999). New developments in the pathogenesis and treatment of steroid-induced osteoporosis. J Bone Miner Res.

[5] Waljee AK, Rogers MA, Lin P, Singal AG, Stein JD, Marks RM (2017). Short term use of oral corticosteroids and related harms among adults in the United States: population based cohort study. Bmj.

[6] McEvoy CE, Ensrud KE, Bender E, Genant HK, Yu W, Griffith JM (1998). Association between corticosteroid use and vertebral fractures in older men with chronic obstructive pulmonary disease. Am J Respir Crit Care Med.

[7] Wang L, Heckmann BL, Yang X, Long H (2019). Osteoblast autophagy in glucocorticoid-induced osteoporosis. J Cell Physiol.

[8] Canalis E, Bilezikian JP, Angeli A, Giustina A (2004). Perspectives on glucocorticoid-induced osteoporosis. Bone..

[9] Kim HJ, Zhao H, Kitaura H, Bhattacharyya S, Brewer JA, Muglia LJ (2006). Glucocorticoids suppress bone formation via the osteoclast. J Clin Invest.

[10] Vasikaran S, Cooper C, Eastell R, Griesmacher A, Morris HA, Trenti T (2011). International Osteoporosis Foundation and International Federation of Clinical Chemistry and Laboratory Medicine position on bone marker standards in osteoporosis. Clin Chem Lab Med.

[11] Bowden SA, Akusoba CI, Hayes JR, Mahan JD (2016). Biochemical markers of bone turnover in children with clinical bone fragility. J Pediatr Endocrinol Metab.

[12] Angeli A, Guglielmi G, Dovio A, Capelli G, de Feo D, Giannini S (2006). High prevalence of asymptomatic vertebral fractures in post-menopausal women receiving chronic glucocorticoid therapy: a cross-sectional outpatient study. Bone..

[13] Laan RF, van Riel PL, van de Putte LB, van Erning LJ, van't Hof MA, Lemmens JA (1993). Low-dose prednisone induces rapid reversible axial bone loss in patients with rheumatoid arthritis. A randomized, controlled study. Ann Intern Med.

[14] Singh D, Agusti A, Anzueto A, Barnes PJ, Bourbeau J, Celli BR, et al. Global Strategy for the Diagnosis, Management, and Prevention of Chronic Obstructive Lung Disease: The GOLD Science Committee Report 2019. Eur Respir J. 2019;53(5):1900164. 10.1183/13993003.00164-2019. 10.1183/13993003.00164-2019 30846476

[15] Ton FN, Gunawardene SC, Lee H, Neer RM. Effects of low-dose prednisone on bone metabolism. J Bone Miner Res. 2005;20(3):464–70. 10.1359/JBMR.041125. 10.1359/JBMR.041125 15746991

[16] Sivapalan P, Moberg M, Eklof J, Janner J, Vestbo J, Laub RR, et al. A multi-center randomized, controlled, open-label trial evaluating the effects of eosinophil-guided corticosteroid-sparing therapy in hospitalised patients with COPD exacerbations - the CORTICO steroid reduction in COPD (CORTICOCOP) study protocol. BMC Pulm Med. 2017;17(1):114. 10.1186/s12890-017-0458-7. 10.1186/s12890-017-0458-7 PMC5558695 28810909

[17] Sivapalan P, Lapperre TS, Janner J, Laub RR, Moberg M, Bech CS, et al. Eosinophil-guided corticosteroid therapy in patients admitted to hospital with COPD exacerbation (CORTICO-COP): a multicentre, randomised, controlled, open-label, non-inferiority trial. Lancet Respir Med. 2019;7(8):699–709. 10.1016/S2213-2600(19)30176-6. 10.1016/S2213-2600(19)30176-6 31122894

[18] Matthew J, Gurka LJE, Nylander-French L. Testing transformations for the linear mixed model. Comput Statist Data Anal. 2007;51(9):4297–307.

[19] Lu K (2010). On efficiency of constrained longitudinal data analysis versus longitudinal analysis of covariance. Biometrics..

[20] Korczowska I, Lacki JK (2005). Changes in certain biochemical markers of bone turnover in rheumatoid arthritis patients treated with short-term low dose glucocorticosteroids. Przegl Lek.

[21] Wislowska M, Jakubicz D, Stepien K, Cicha M (2009). Serum concentrations of formation (PINP) and resorption (Ctx) bone turnover markers in rheumatoid arthritis. Rheumatol Int.

[22] Censi S, Manso J, Pandolfo G, Franceschet G, Cavedon E, Zhu YH (2019). Bone turnover markers, BMD and TBS after short-term, high-dose glucocorticoid therapy in patients with Graves' orbitopathy: a small prospective pilot study. J Endocrinol Investig.

[23] Bornefalk E, Dahlen I, Michaelsson K, Ljunggren Ö, Ljunghall S (1998). Age-dependent effect of oral glucocorticoids on markers of bone resorption in patients with acute asthma. Calcif Tissue Int.

[24] Naylor KE, Jacques RM, Paggiosi M, Gossiel F, Peel NF, McCloskey EV (2016). Response of bone turnover markers to three oral bisphosphonate therapies in postmenopausal osteoporosis: the TRIO study. Osteoporos Int.

[25] Miller PD, Pannacciulli N, Malouf-Sierra J, Singer A, Czerwinski E, Bone HG (2020). Efficacy and safety of denosumab vs. bisphosphonates in postmenopausal women previously treated with oral bisphosphonates. Osteoporos Int.

[26] Christgau S, Bitsch-Jensen O, Hanover Bjarnason N, Gamwell Henriksen E, Qvist P, Alexandersen P (2000). Serum CrossLaps for monitoring the response in individuals undergoing antiresorptive therapy. Bone..

[27] Hygum K, Starup-Linde J, Harslof T, Jorgensen NR, Hartmann B, Holst JJ (2019). The diurnal variation of bone formation is attenuated in adult patients with type 2 diabetes. Eur J Endocrinol.

[28] Qvist P, Christgau S, Pedersen BJ, Schlemmer A, Christiansen C (2002). Circadian variation in the serum concentration of C-terminal telopeptide of type I collagen (serum CTx): effects of gender, age, menopausal status, posture, daylight, serum cortisol, and fasting. Bone.

