# Supplementary material for: Bone turnover biomarkers in COPD patients randomized to either a regular or shortened course of corticosteroids: a substudy of the randomized controlled CORTICO-COP trial
Source: Respir Res. 2020 Oct 12;21:263. doi: 10.1186/s12931-020-01531-9 (PMC7552546; doi:10.1186/s12931-020-01531-9)
Supplement: Supplementary file 1 — Additional file 1. [file 12931_2020_1531_MOESM1_ESM.docx]

**Supplementary Material**

**Bone turnover in COPD patients randomized to either a regular or shortened course of corticosteroids**

**Pradeesh Sivapalan^1,2^, Niklas R. Jørgensen^3,4^, Alexander G. Mathioudakis^5,6^, Josefin Eklöf^1^, Therese Lapperre^7^, Charlotte Suppli Ulrik^8^, Helle F. Andreassen^7^, Karin Armbruster^1^, Praleene Sivapalan^1^, Julie Janner^7^, Nina Godtfredsen^8^, Ulla M. Weinreich^9^, Thyge L. Nielsen^10^, Niels Seersholm^1^, Torgny Wilcke^1^, Philipp Schuetz^11,12^, Tobias W. Klausen^13^, Kristoffer Marså^14^, Jørgen Vestbo^5,6^, Jens-Ulrik Jensen^1,4^.**

^1^Section of Respiratory Medicine, Department of Medicine, Herlev and Gentofte Hospital, University of Copenhagen, Hellerup, Denmark; ^2^Department of Internal Medicine, Zealand Hospital, University of Copenhagen, Roskilde, Denmark; ^3^Department of Clinical Biochemistry, Copenhagen University Hospital Rigshospitalet, Copenhagen, Denmark; ^4^Department of Clinical Medicine, Faculty of Health and Medical Sciences, University of Copenhagen, Copenhagen, Denmark; ^5^The North West Lung Centre, Wythenshawe Hospital, Manchester University NHS Foundation Trust, Manchester, UK; ^6^Division of Infection, Immunity and Respiratory Medicine, School of Biological Sciences, The University of Manchester, Manchester Academic Health Science Centre, UK; ^7^Department of Respiratory Medicine, Bispebjerg University Hospital, Copenhagen, Denmark; ^8^Department of Respiratory Medicine, Amager and Hvidovre University Hospital, Copenhagen, Denmark; ^9^Department of Respiratory Diseases, Aalborg University Hospital, Aalborg, Denmark; The Clinical Institute, Aalborg University, Aalborg, Denmark; ^10^Department of Respiratory and Infectious Diseases, Frederiksund and Hillerød Hospital, University of Copenhagen, Denmark; ^11^Medical University Department, Kantonsspital Aarau, 5001 Aarau, Switzerland; ^12^Faculty of Medicine, University of Basel, 4001 Basel, Switzerland; ^13^Clinical Research Unit, Department of Hematology, Herlev Hospital, Herlev, Denmark; ^14^Palliative Medicine Section Unit, Herlev and Gentofte Hospital;

As a post hoc analysis, we looked at whether CTX and P1NP biomarkers were affected by the cumulative dose of corticosteroids. We excluded patients taking bisphosphonates, denosumab or synthetic human parathyroid within the 12 months before inclusion.

**Table 1S. Linear regression model**

|  | Slope pr 100 mg | 95% CI | P | R^2^ |
| --- | --- | --- | --- | --- |
| P1NP, 30 days | -0.024 | -0.049; -0.000 | 0.047 | 0.02 |
| P1NP, 90 days | -0.018 | -0.028; -0.009 | 0.002 | 0.09 |
| CTX, 30 days | -0.030 | -0.068; 0.007 | 0.12 | 0.01 |
| CTX, 90 days | -0.006 | -0.021; 0.009 | 0.42 | 0.00 |

For P1NP we observed that there was a linear decrease in P1NP for increasing corticosteroids dose. This was most evident for cumulative dose of corticosteroids within 30 days.

For CTX we observed no significant change for cumulative dose of corticosteroids for 30 days and 90 days of follow-up.

**Figure 1S. Change in log P1NP from baseline to 1- and 3-month follow-up for cumulative dose of corticosteroids**

P1NP is a marker for bone formation. There was a linear decrease in P1NP levels at both 1 month (*p* = 0.047) and 3 months (*p* = 0.002) for cumulative dose of corticosteroids.


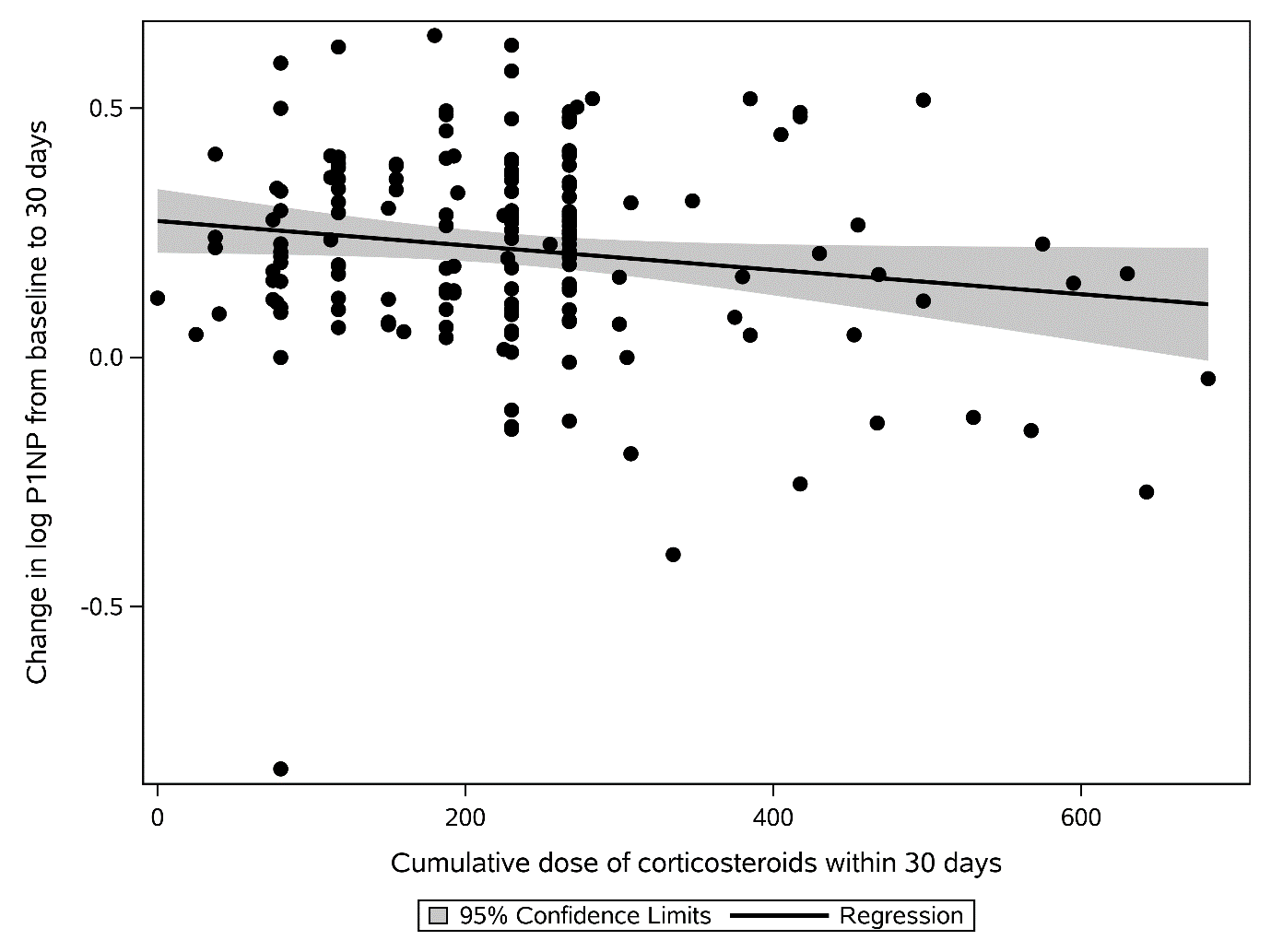


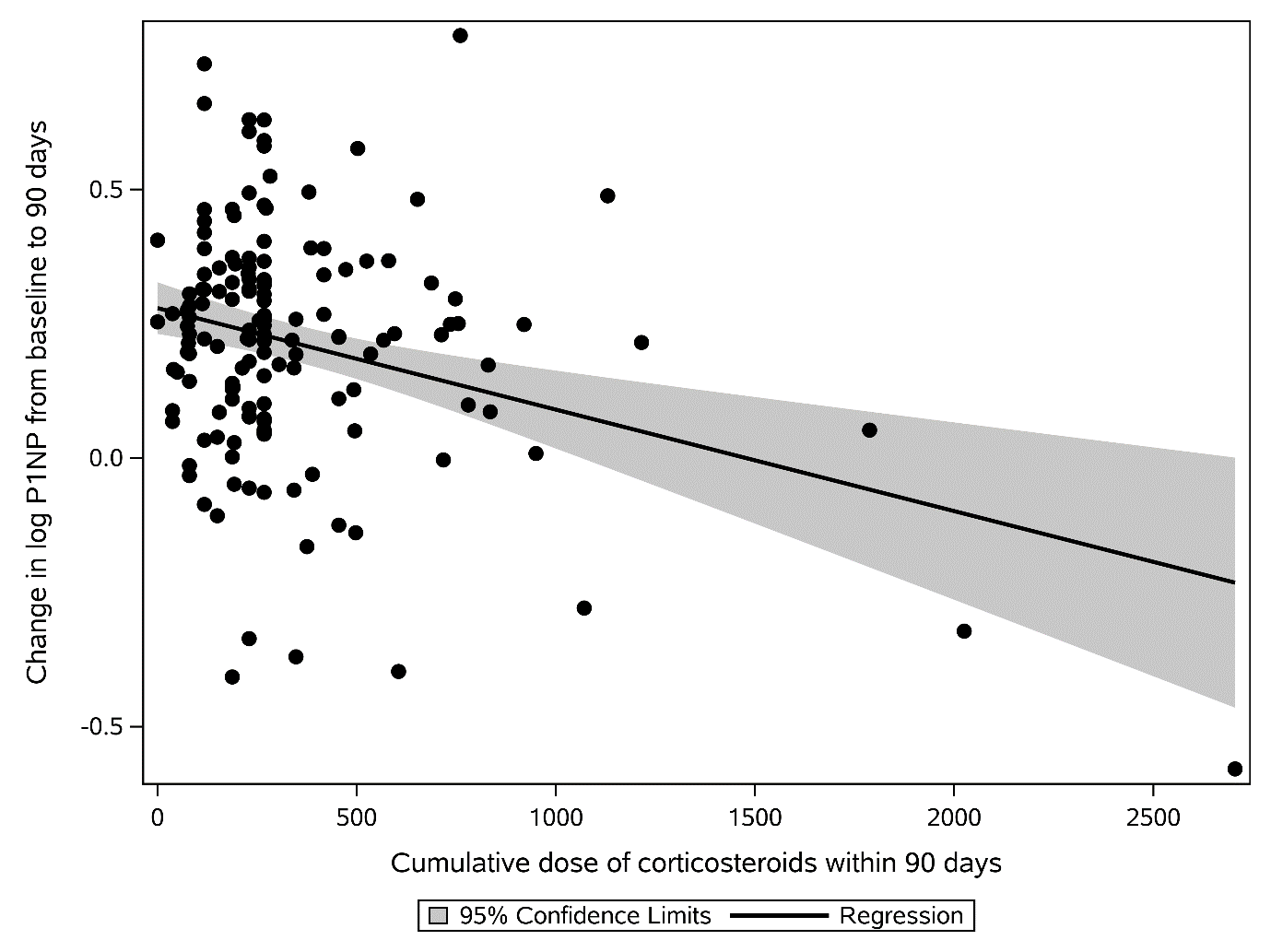


**Figure 2S. Change in log CTX levels from baseline to 1- and 3-month follow-up**

CTX is a marker for bone resorption. No change for CTX levels for cumulative dose of corticosteroids was observed at 30 days (*p* = 0.12) and 90 days of follow-up (*p* = 0.42).


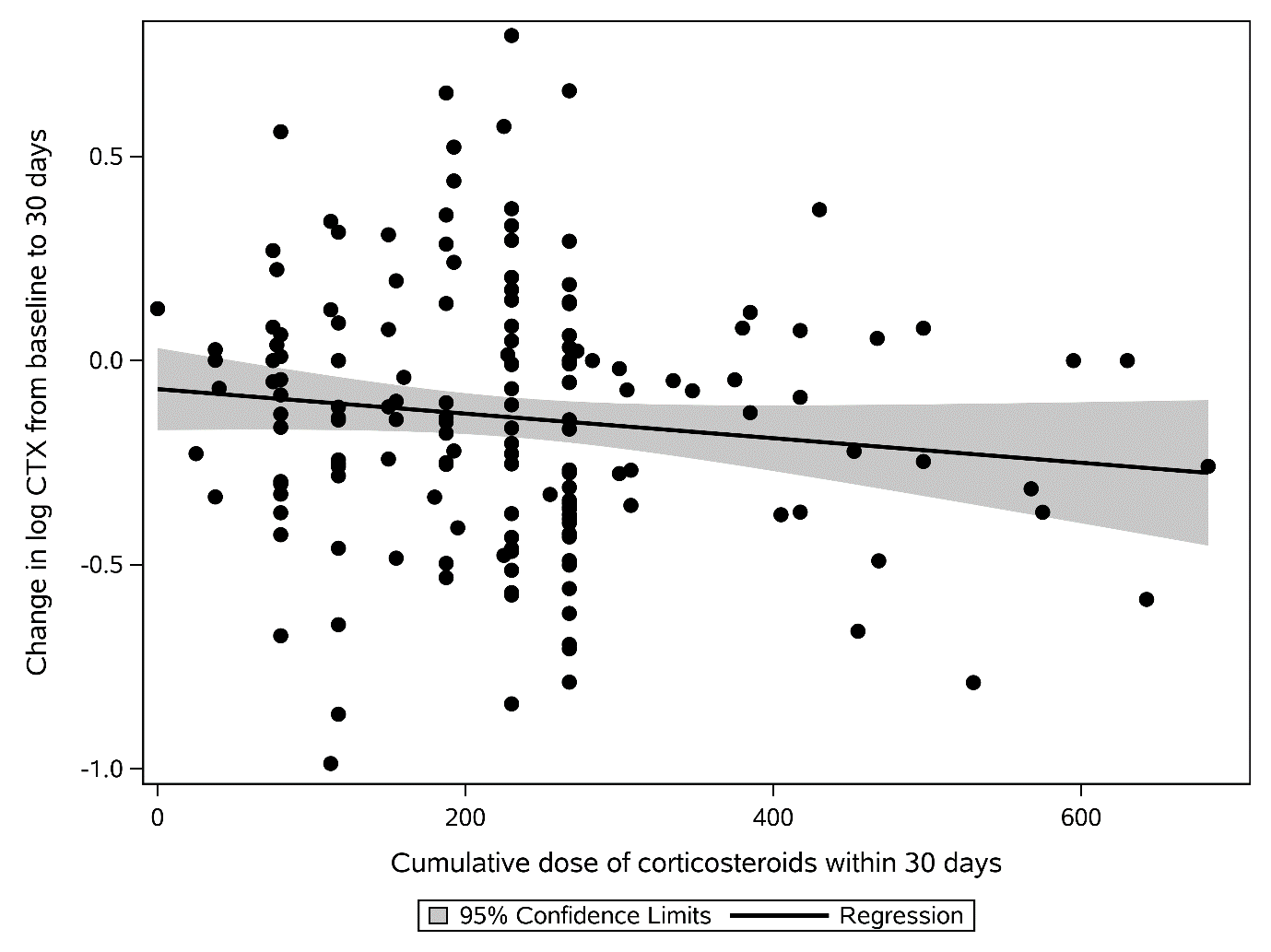


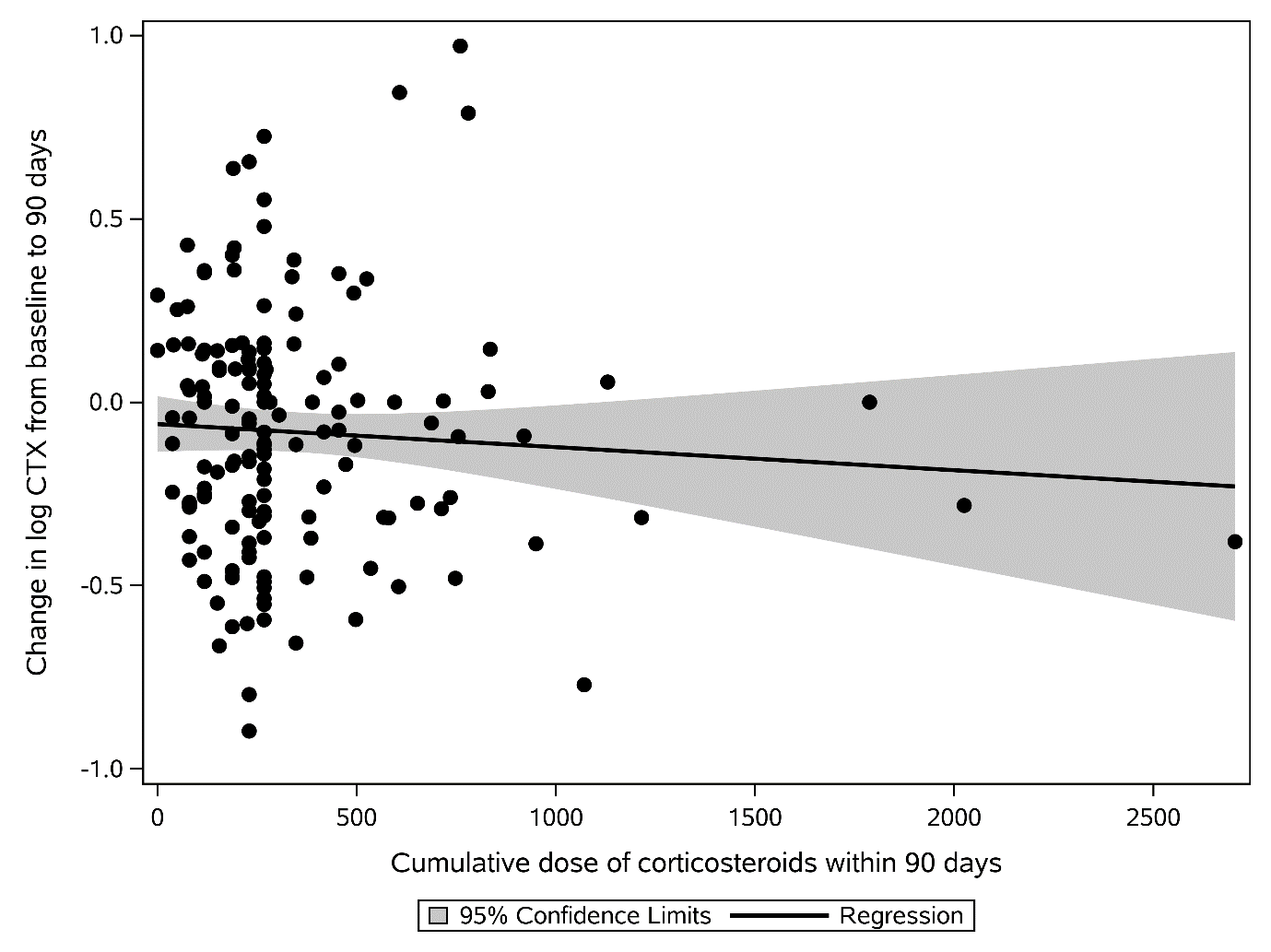


**Table 2S. Change in BTMs in low doses group compared to high doses for ICS users vs non ICS users.**

|  | Adjusted diff. 1M  (95% CI) | P | Adjusted diff. 3M  (95% CI) | P |
| --- | --- | --- | --- | --- |
| P1NP, No ICS | -0.8 (-16.2; 17.4) | 0.92 | -14.9 (-29.6; 2.7) | 0.092 |
| P1NP, ICS | -3.2 (-18.0; 14.2) | 0.70 | 0.8 (-16.1; 20.7) | 0.95 |
| CTX, No ICS | 17.3 (-10.4; 53.7) | 0.24 | 10.5 (-18.4; 49.5) | 0.52 |
| CTX, ICS | 3.7 (-16.6; 29.0) | 0.74 | 37.4 (10.2; 71.4) | 0.005 |

**Figure 3S. For both CTX and P1NP a lambda value at 0.0 performed well indicating log transformation is suitable for these outcomes. The figure shows Log-likelihood dependent on lambda values. For both outcomes the maximum likelihood is close to a lambda value at 0 equal to log transformation.**


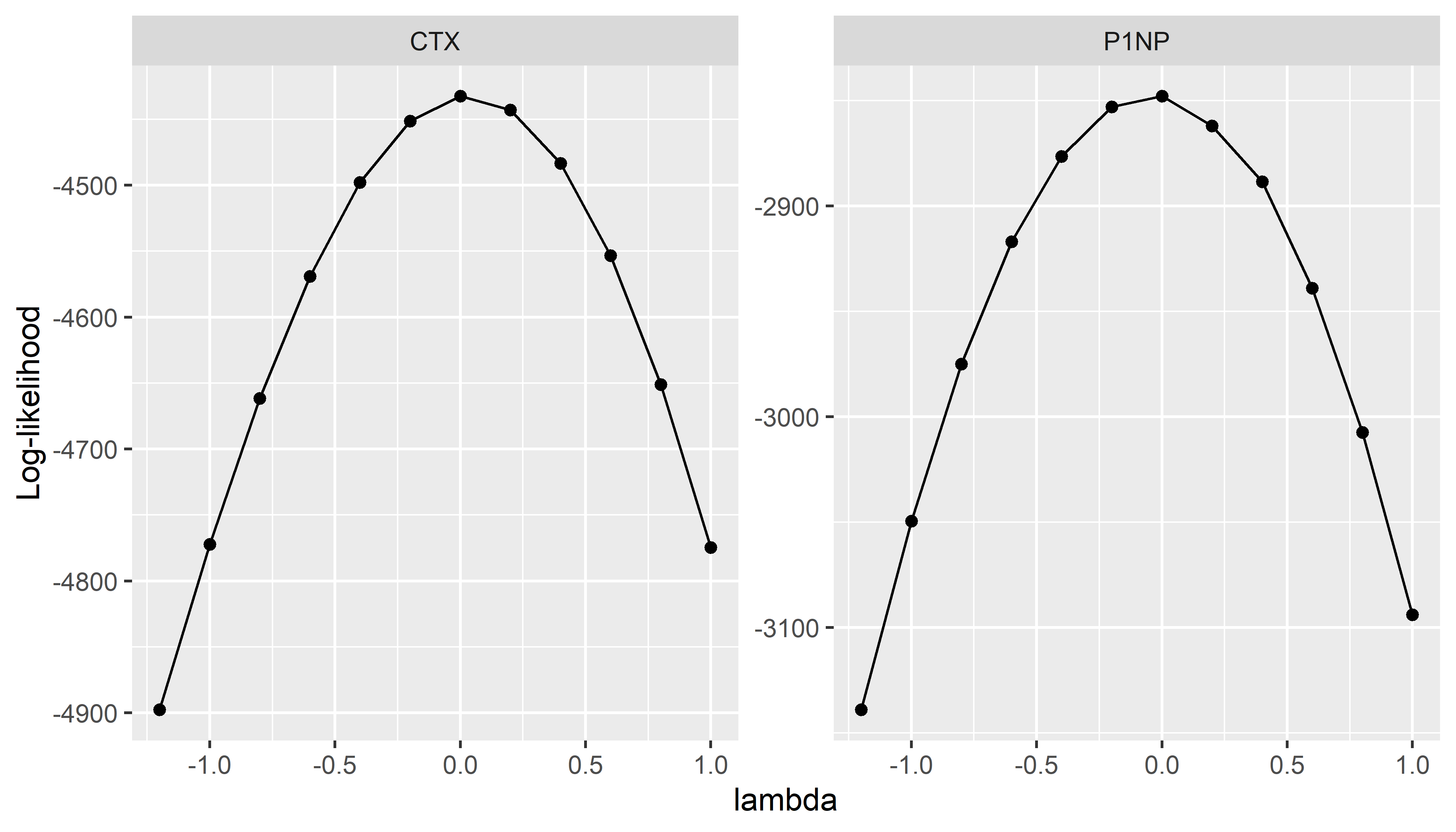


**Table 3S**

**Median time and the frequency of fasting blood samples in the two treatment groups**

|  | Blood test time day 2 | Blood test time day 30 | Blood test time day 90 |
| --- | --- | --- | --- |
| Time, median (IQR) | 06:52 (06:13 - 07:25) | 10:11 (09:00 - 12:03) | 10:30 (9:25 - 12:36) |
| Frequency of fasting blood samples (before 8 a.m. ) in the two treatment groups | *Low dose:*  118/142 = 83.1 %  High dose: 129/144 = 89.6%, *p* = 0.11 | *Low dose:*  7/76 = 3.8%  High dose:  13/89 = 7%, *p* = 0.35 | *Low dose:*  3/71 = 1.9%  High dose: 10/74 = 6.3%, *p* = 0.07 |
| Comparing time of blood samples in the two treatment groups: | *Low dose:*  *6:52 (6:11-7:25)*  *High dose:*  *6:58 (6:19-7:21)*  *P* = 0.92 | *Low dose:*  *10:15 (9:20-11:59)*  *High dose:*  *10:00 (8:47-12:05)*  *P* = 0.56 | *Low dose:*  *10:45 (9:30-12:00)*  *High dose:*  *10:28 (9:09-12:57)*  *P* = 0.85 |
